# Does Cystatin C have a role as metabolic surrogate in peritoneal dialysis beyond its association with residual renal function?

**DOI:** 10.1590/2175-8239-JBN-2019-0007

**Published:** 2019-12-02

**Authors:** Carla Leal Moreira, Liliana Cunha, Sofia Correia, Filipa Silva, Ana Castro, Joana Tavares, Maria João Carvalho, José Carlos Oliveira, Olívia Santos, António Cabrita, Anabela Rodrigues

**Affiliations:** 1 Centro Hospitalar do Porto, Hospital de Santo António, Departamente de Nefrologia, Porto, Portugal.; 2 Centro Hospitalar do Porto, Hospital de Santo António, Departamento de Patologia,Porto, Portugal.

**Keywords:** Cystatin C, Cardiovascular Diseases, Peritoneal Dialysis, Cistatina C, Doenças Cardiovasculares, Diálise Peritoneal

## Abstract

**Introduction:**

It has been suggested that cystatin C levels are modified by obesity and inflammation. Furthermore, cystatin C has been associated with cardiovascular events and mortality outcomes.

**Aim:**

To study the association of cystatin C with the metabolic profile and cardiovascular disease of peritoneal dialysis patients.

**Methods:**

Data collected included clinical, laboratorial, and multifrequency bioimpedance assessment of 52 stable peritoneal dialysis patients. Minimal residual renal function was defined as > 2mL/min/1.73m^2^.

**Results:**

Serum cystatin C was not significantly associated with peritoneal or urinary cystatin C excretion. Negative correlation of cystatin C with normalized protein catabolic rate (rho -0.33, *p =* 0.02) and a trend towards positive correlation with relative body fat (rho 0.27, *p =* 0.05) were not independent from residual renal function. Cystatin C was not significantly associated with cardiovascular disease (*p =* 0.28), nor with glycated hemoglobin (*p =* 0.19) or c-reactive protein (*p =* 0.56). In the multivariate model, both age and diabetes were the strongest predictors of cardiovascular disease (odds ratio 1.09, *p =* 0.029 and odds ratio 29.95, *p =* 0.016, respectively), while relative body fat was negatively associated with cardiovascular disease (*p =* 0.038); neither cystatin C (*p* = 0.096) nor minimal residual renal function (*p* = 0.756) reached a significant association with cardiovascular disease.

**Conclusions:**

In this group of peritoneal dialysis patients, cystatin C did not correlate with the metabolic or inflammatory status, nor cardiovascular disease, after adjustment for residual renal function.

## RESUMO

**Introdução:**

Tem sido sugerido que os níveis de cistatina C são modificados pela obesidade e inflamação. Além disso, a cistatina C tem sido associada a eventos cardiovasculares e desfechos de mortalidade.

**Objetivo:**

Estudar a associação da cistatina C com o perfil metabólico e doença cardiovascular de pacientes em diálise peritoneal.

**Métodos:**

Os dados coletados incluíram avaliação clínica, laboratorial e de bioimpedância múltipla de 52 pacientes estáveis em diálise peritoneal. A função renal residual mínima foi definida como > 2mL/min/1,73m^2^.

**Resultados:**

A cistatina C sérica não esteve significativamente associada à excreção peritoneal ou urinária. A correlação negativa da cistatina C com a taxa catabólica protéica normalizada (rho -0,33, *p =* 0,02) e uma tendência de correlação positiva com a gordura corporal relativa (rho 0,27, *p =* 0,05) não foram independentes da função renal residual. A cistatina C não se associou significativamente à doença cardiovascular (*p =* 0,28), nem com hemoglobina glicada (*p =* 0,19) ou proteína C reativa (*p =* 0,56). No modelo multivariado, idade e diabetes foram os mais fortes preditores de doença cardiovascular (razões de probabilidade 1,09, *p =* 0,029 e 29,95, *p =* 0,016, respectivamente) enquanto a gordura corporal relativa se associou negativamente à doença cardiovascular (*p =* 0,038). A cistatina C não se associou significativamente com doença cardiovascular (*p =* 0,096), tampouco a função residual mínima (*p =* 0,756).

**Conclusão:**

Neste grupo de pacientes em diálise peritoneal, a cistatina C não se correlacionou com o estado metabólico ou inflamatório, nem com doença cardiovascular, após ajuste para função renal residual.

## INTRODUCTION

Cystatin C (CysC) has been proposed to be strongly associated with cardiovascular events and mortality outcomes. Furthermore, some authors report that CysC-based estimated glomerular filtration rate (eGFR) is associated with all-cause mortality and cardiovascular events independent of both measured or creatinine-based eGFR^1^^-^ 3.

Cystatin C is a low molecular weight (13.3 kDa) protein that is freely filtered, almost totally reabsorbed and metabolized in proximal tubule of the nephron^4^. It is also known for its constant production (by nucleated cells), except in the cases of steroid users, obesity, inflammation, and thyroid malfunction. Additionally, it has been found to be poorly influenced by body mass composition, diet, gender and ethnic group, supporting the use of CysC-based equations for GFR estimation in pre-dialysis patients^5^^-^ 7.

Moreover, serum CysC accuracy in estimating residual renal function has been studied in peritoneal dialysis (PD) patients^8^^-^ 10 and its potential role beyond GFR prediction has been also investigated. CysC has been suggested to be an independent predictor of mortality, cardiovascular events, and end-stage renal disease (ESRD)^11^^,^ 12. In kidney transplant recipients, CysC was found to be an independent predictor of cardiovascular events, mortality, and kidney failure^13^. Furthermore, in patients with negligible GFR (hemodialysis and peritoneal dialysis patients) CysC maintained its predictive power for adverse outcomes^14^.

We hypothesized that CysC has a role beyond GFR estimation. Therefore, we conducted a cross-sectional study to evaluate CysC ability to predict metabolic and major cardiovascular disease (CVD) in peritoneal dialysis (PD) patients.

## METHODS

We performed a cross-sectional study enrolling 52 patients followed in our outpatient PD clinic at Centro Hospitalar do Porto (CHP) from October 2016 until January 2017. Patients were included if clinically stable and on PD therapy for at least 3 months, and excluded if acutely ill. The clinical and laboratorial data were collected as part of the national quality control and approved by national data protection committee. A single assessment of serum, urinary, and peritoneal effluent CysC levels was determined by an automated particle-enhanced nephelometric immunoassay (Dimension Vista 500, Siemens Healthcare). Other laboratory measurements were performed in the laboratories of the CHP using standardized and automated methods. Lean tissue mass (LTM), fat mass, body cell mass (BCM), and relative overhydration (rel.OH) were assessed by the multifrequency bioimpedance method using the Body Composition Monitor (BCM, Fresenius Medical Care, Bad Homburg, Germany). The bioimpedance method applied was validated by isotope dilution methods^15^, by accepted reference body composition methods^16^^,^ 17, and by extensive clinical assessment of the hydration state^18^. Body composition assessment was obtained with full abdomen. The patient was weighed after draining out peritoneal effluent and thereafter 2 liters of peritoneal solution were instilled. All BCM measurements were performed by a trained nurse with the patient in supine position on a non-conductive bed, in resting conditions. Two non-recyclable electrode strips were placed on the dorsal surface of the wrist and foot on one side of the body and connected to the BCM device.

Patients’ past medical history, including major cardiovascular events, medication, PD modality, dialysis adequacy, daily exchange volume, and ultrafiltration were collected. Self-reported major cardiovascular disease at baseline included prior myocardial infarction, coronary artery revascularization, stroke, or carotid arterial revascularization. Adequacy of dialysis was calculated from 24-hour urine and dialysate collection. Weekly Kt/V was determined using standard methods^19^. Residual renal function (RRF) was assessed simultaneously with CysC evaluation, using the average clearance of urea and creatinine (CrUCL) in 24-h urine collection, as described elsewhere^20^. Patients were considered to have minimal RRF if CrUCL > 2mL/min/1.73m^2^. A panel of metabolic biomarkers including HOMA-IR (homeostasis model assessment insulin resistance index), leptin, lipid profile, and insulin was obtained on the same day of peritoneal equilibration test. Dietary protein intake was estimated from protein catabolic rate normalized to actual body weight (nPCR) using the PD Adequest software (Baxter Healthcare Corporation, Deerfield, IL). All patients were treated with low-glucose degradation product solutions; 3.86% glucose exchanges were not routinely used. Icodextrin was used in 27 (51.9%) patients.

The primary outcome was the correlation of CysC with cardiovascular disease. Secondary outcomes were CysC correlation with the metabolic panel and bioimpedance measurements. The results are reported as the mean ± standard deviation or median and interquartile range (IQR) according to normality distribution. The association between CysC and other variables was estimated using Spearman’s rank correlation for continuous variables and Wilcoxon rank-sum test for categorical data. Chi-square test was applied to categorical variables. We assessed the association of CysC level with CVD and metabolic parameters using adjusted logistic and linear regression, respectively. RRF was the only confounding variable included in the linear regression model of CysC correlation with metabolic parameters. Clinically significant and statistically significant variables were included in the adjusted model of CVD prediction. Measures of association are reported as odds ratio (OR) and 95% confidence intervals (CI). A *p* value < 0.05 was considered statistically significant. Statistical analysis was performed in Stata, version 14.0 (StataCorp LP^®^).

## RESULTS

Fifty-two patients were eligible for this study, of which 52% (n = 27) were male patients, mean age was 51.5 ± 13.8 years. Time on PD was 21.6 (13.1 -50.3) months and most patients received continuous ambulatory PD (n = 30, 58%). The prevalence of diabetes and hypertension was 11.5% (n = 6) and 94% (n = 49), respectively; 50% (n = 26) had medicated dyslipidemia. Fifteen (19%) patients reported current or past smoking habits. Twelve patients reported previous history of cardiovascular disease, 10 patients (19%) were previously diagnosed with ischemic heart disease (IHD), and four patients (8%) with a prior stroke. The baseline characteristics of this cohort are listed on Table 1.

**Table 1:** Baseline characteristics of prevalent peritoneal dialysis patients

Variables	N
Number of patients	52
Age (years), mean (SD)	51.5 ± 13.8
Gender (males)	27 (52%)
Hypertension	49 (94%)
Diabetes Mellitus	6 (11.5%)
Dyslipidemia	26 (50%)
Smoking status	
Non-smoker	37 (71%)
Previous smoker	9 (17%)
Current smoker	6 (1%)
Chronic kidney disease etiology	
Diabetic nephropathy	3 (6%)
ADPKD	3 (6%)
Chronic GN	30 (58%)
Nephrosclerosis	1 (2%)
Other	15 (29%)
Ischemic heart disease (%)	10 (19%)
Cerebrovascular disease (%)	4 (8%)
Charlson Comorbidities Index, median (IQR)	3 (2, 4)
Total time on Renal Replacement Therapy (months)	33.1 (16.0, 77.5)
Time on PD duration (months)	21.6 (13.1, 50.3)
PD modality	
APD	22 (42%)
CAPD	30 (58%)
Weekly Kt/V	2.0 (1.8, 2.4)
Weekly CrCL (L/1.73m^2^)	72.5 (56.7, 95.1)
Serum CysC (mg/L)	5.9 (4.7, 6.9)
Serum Creatinine (mg/dL)	8.6 (6.8,12.2)
CrUCL (mL/min/1.73m^2^)	4.0 (2.4, 8.3)
Hemoglobin (g/dL)	10.90 (9.75, 11.60)
Metabolic status	
HbA1C (%)	5.5 (5.3, 5.9)
Glucose (mg/dL)	90 (81, 105.5)
Insulin (UI/mL)	11.2 (7.6, 17)
HOMA-IR	2 (2, 4)
Leptin (ng/mL)	48.4 (15.4, 84.9)
Total Cholesterol (mg/dL)	173 (151, 196)
LDL-cholesterol (mg/dL)	96.5 (73.5, 121)
HDL- cholesterol (mg/dL)	41 (33.5, 56)
Triglyceride (mg/dL)	161 (98, 209)
nPCR (g/Kg)	1.0 (0.9, 1.3)
Albumin (g/dL)	3.9 (3.8, 4.2)
b2-M (mg/L)	23.5 (17.5, 33.1)
C-Reactive Protein (mg/L)	3.2 (0.7, 9.3)
Mineral bone disease	
Intact parathyroid hormone (pg/mL)	521.4 (303.5, 665.5)
Calcium (mmol/L)	2.2 (2.0; 2.3)
Phosphate (mmol/L)	1.5 (1.3, 1.8)

Data presented as mean ± SD, median (interquartile range), or percent frequency, as appropriate. ADPKD: autosomal dominant polycystic kidney disease; GN: glomerulonephritis; PD: peritoneal dialysis; APD: automatic peritoneal dialysis; CAPD: continuous ambulatory peritoneal dialysis; CrCL: creatinine clearance; CysC: cystatin C; CrUCL: clearance of urea and creatinine in 24-h urine collection; HbA1c: glycated hemoglobin; LDL: low density lipoprotein; HDL: high density lipoprotein; nPCR: normalized protein catabolic rate; β 2-M: beta-2-microglobulin.

The median serum level of CysC was 5.9 (4.7 - 6.9) mg/L, with the median level of serum creatinine being 8.6 (6.8 -12.2) mg/dL. Twelve patients (23.1%) presented a daily urine output < 200 mL. Median RRF was 4.0 (2.4 - 8.3) mL/min/1.73m^2^. All participants had adequate dialysis clearances according to the weekly Kt/V and creatinine clearance (CrCL). The median weekly Kt/V was 2.0 (1.8 - 2.4). Complementary laboratorial data on mineral bone disease and metabolic parameters are listed on Table 1.

The body composition was analyzed providing additional data regarding hydration and nutritional status (Table 2).

**Table 2:** Bioimpedance assessment

Factors	Value
Weight (kg)	71.8 (62.8, 82.2)
Relative OH (%)	5.8 (0.4, 11.8)
ECW (L)	17.2 (14.2, 18.9)
ICW (L)	18.9 (15, 21.5)
BMI (kg/m^2^)	25.4 (23, 29.1)
LTM (kg)	36.9 (29.6, 46.4)
Relative LTM (%)	51.9 (41.7, 60)
Fat (kg)	23.5 (17, 32.3)
Relative Fat (%)	33.2 (28.1, 41.6)
Body Cell Mass (kg)	19.8 (15.6, 26.3)

Data presented as median (interquartile range). OH: overhydration. ECW: extracellular weight; ICW: intracellular weight; BMI: body mass index. LTM: lean total mass.

Patients with IHD (6.5 vs 5.7 mg/L, *p* = 0.44) and cerebrovascular disease (6.2 vs 5.7 mg/L, *p* = 0.41) had a trend for higher levels CysC, without reaching statistical significance, Table 3. When the composite end-point of CVD was considered, serum CysC levels difference remained statistically non-significant (6.5 vs. 5.6 mg/L, *p* = 0.28).

**Table 3:** CysC levels according to cardiovascular risk factors and major cardiovascular disease

Factors	Median CysC (mg/L)	*p*-value^a^
Gender (male/female)	6.1 vs 5.6	0.77
Hypertension (Y/N)*	5.8 vs 6.4	0.98
Diabetes Mellitus (Y/N)*	5.2 vs 5.9	0.38
Previous or current smoker status (Y/N)*	6.6 vs 5.6	0.10
Dyslipidemia (Y/N)*	5.7 vs 6.2	0.22
Ischemic heart disease (Y/N)*	6.5 vs 5.7	0.44
Cerebrovascular disease (Y/N)*	6.2 vs 5.7	0.41

* Y/N (Yes/No).

a A *p* value < 0.05 was considered statistically significant.

Patients receiving automatic PD correlated positively with CysC (rho: 2.82, *p* = 0.005), PD duration (rho 0.36, *p* = 0.007) and weekly peritoneal CrCL (rho: 0.43, *p* = 0.002), but inversely with weekly renal CrCL (rho: -0.70, *p* < 0.0001), consistent with its correlation with RRF. CysC levels did not correlate significantly with the lipid profile, HOMA-IR score, or glycated hemoglobin, but correlated negatively with nPCR (rho: -0.33, *p* = 0.02) and hemoglobin (rho: -0.29, *p* = 0.03), Table 4.

**Table 4:** Correlation analysis of metabolic and inflammatory variables associated with serum CysC level

Variables	Correlation coefficient	*p*-value*
Age	-0.25	0.08
Charlson Comorbidities Index	-0.19	0.16
PD modality	2.82	**0.005**
PD duration	0.36	**0.007**
Creatinine dialysate-to-plasma ratio	-0.02	0.88
Weekly renal CrCL	-0.70	**< 0.0001**
Weekly peritoneal CrCL	0.43	**0.002**
Protein loss	0.11	0.42
Urinary CysC excretion	0.08	0.62
Peritoneal CysC excretion	-0.03	0.83
Insulin	-0.18	0.27
Leptin	0.12	0.48
HOMA-IR score	-0.16	0.33
Total cholesterol	0.19	0.25
HDL-cholesterol	-0.14	0.36
Albumin	0.14	0.34
HbA1c	-0.21	0.19
nPCR	-0.33	**0.02**
C-Reactive Protein	0.08	0.56
Ferritin	0.09	0.51
Hemoglobin	-0.29	**0.03**

PD: peritoneal dialysis; CrCL: creatinine clearance; CysC: cystatin C; HOMA-IR: homeostasis model assessment insulin resistance index; HDL: high density lipoprotein; HbA1c: glycated hemoglobin; nPCR: normalized protein catabolic rate.

* A *p* value < 0.05 was considered statistically significant.

Regarding the bioimpedance evaluation, CysC correlated negatively with the relative total lean mass (rho: -0.29, *p* = 0.047) and positively with the relative fat (rho: 0.27, *p* = 0.05), Table 5.

**Table 5:** Correlation analysis of body composition monitoring variables with serum CysC level

Variables	Correlation coefficient	*p*-value*
Total weight (kg)	0.06	0.64
Relative OH (%)	0.07	0.63
Non-hydrated weight (kg)	0.06	0.70
ECW (L)	- 0.06	0.67
ICW (L)	- 0.20	0.16
BMI (kg/m^2^)	0.21	0.14
LTM (kg)	- 0.22	0.12
Relative LTM (%)	- 0.29	0.047
Total fat (kg)	0.22	0.13
Relative fat (%)	0.27	0.05
Body cell mass (kg)	- 0.21	0.13

OH: overhydration; ECW: extracellular weight; ICW: intracellular weight; BMI: body mass index; LTM: lean total mass.

* A *p* value < 0.05 was considered statistically significant.

In the linear regression model adjusted for RRF, CysC no longer correlated with lean mass, fat body composition, or nPCR. In the multivariate model adjusted for minimal RRF, CysC, age, and diabetes, both age and diabetes were the strongest predictors for CVD (OR: 1.09, 95%CI: 1.01-1.17, *p* = 0.029 and OR: 29.95, 95%CI: 1.87-479.98, *p* = 0.016, respectively), with relative fat percentage associated negatively with CVD (OR: 0.91, 95%CI: 1.01-1.17, *p* = 0.038); CysC did not reach a statistically significant positive association with CVD (OR: 2.65, 95%CI: 0.84-8.38, *p* = 0.096), neither did minimal RRF (*p* = 0.756), Table 6.

**Tabela 6:** Multivariate analysis: adjusted model of predictors for cardiovascular disease

	OR	95% CI	*p* value*
Serum CysC	2.65	0.84-8.37	0.096
RRF	0.65	0.04-9.93	0.756
Relative fat	0.91	0.83-0.99	0.038
Diabetes	29.95	1.87-479.98	0.016
Age	1.09	1.01-1.17	0.029

CysC: cystatin C; OR: odds ratio; CI: confidence intervals; RRF: residual renal function.

* A *p* value < 0.05 was considered statistically significant.

## DISCUSSION

To the best of our knowledge, this is the first study to address CysC correlation with an extensive panel of metabolic biomarkers, bioimpedance metrics, and cardiovascular disease in PD patients.

Cardiovascular disease is the major cause of death in patients with chronic kidney disease, both in pre-dialysis patients and in end-stage renal disease. Moreover, incident PD patients have been shown to have an increased risk of cardiovascular mortality after the first year^21^. Authors have searched for serum biomarkers able to predict CVD and CVD mortality.

CysC has been suggested to be correlated with CVD in patients with normal renal function^5^. CysC independent correlation with CVD was hypothesized in a study where eGFR based on either creatinine or CysC was influenced by traditional cardiovascular risk factors even after adjusting for measured GFR^1^. Tangri et al. findings suggested that CysC was associated with kidney failure, CVD death, and all-cause mortality. In fact, adjustment for GFR strengthened the association of CysC with mortality outcomes in pre-dialysis patients^11^.

Whether CysC represents a marker or has an active role in atherosclerosis process and its pathways remains to be clarified. CysC inhibitory activity is vital for the regulation of normal physiological processes by limiting the potentially highly destructive activity of its target proteases, including cysteine cathepsins. Naour et al. found a significant increase in CysC mRNA expression in omental and subcutaneous adipose tissue and three-fold increase in obese patients^22^. Additionally, epicardial adipose tissue was also found to be positively correlated with serum CysC, leptin, body mass index, and age. On the other hand, when CysC was replaced with eGFR, eGFR showed no significant correlation with epicardial adipose tissue^23^. Others have found circulating CysC to be consistently elevated in obese subjects, independently of reduced eGFR. This finding may be supported by CysC role on regulatory mechanisms engaged to control the pro-atherogenic activity of specific cathepsins^24^. Taken together, these reports favor the pathogenic role of CysC in atherogenesis.

In our study, CysC levels were found to be elevated in older patients, patients with past or present history of smoking, ischemic heart disease, and cerebrovascular disease, though without statistical significance. We also found CysC levels to be correlated with body fat composition, with higher levels of CysC identified in subjects with higher relative fat percentage, and inversely correlated with relative LTM. Conversely, CysC negative correlation with nPCR supports that higher CysC levels correlated with worse nutritional status, however statistical significance was lost once adjustment for RRF was applied. As others have suggested, lower nPCR was associated with poorer nutritional status and increased risk of all-cause mortality in PD patients^25^.

A recent study in PD patients reported that in obese patients, fat tissue index and leptin/adiponectin ratio were correlated with HOMA-IR, independently of glucose absorption, small-solute transport, and time on PD^26^. In our study, we failed to show any statistically significant association of CysC with leptin, insulin resistance score, and glycated hemoglobin. Additionally, we did not find CysC to be associated with inflammatory parameters such as ferritin or c-reactive protein.

In the multivariate model, relative fat body composition was associated with a protective effect of CVD. Indeed, overall higher relative fat percentage could reflect less severe systemic disease and improved nutritious status. However, we did not assess body fat distribution, so whether this relative fat percentage was referring to visceral or subcutaneous fat could not be determined.

A previous study in both PD and hemodialysis patients with minimal RRF found CysC to be associated with adverse cardiovascular and infection events independently of the GFR^14^. Nevertheless, our results did not support a statistically significant association of CysC with cardiovascular disease, independently of RRF.

We acknowledge the limitations of our study, namely its observational and cross-sectional design, so causality cannot be established. Furthermore, we enrolled a relatively small cohort of PD patients, limiting the generalization of our results.

However, it must be emphasized that our study found CysC to be increased in patients with lower renal weekly creatinine clearance, supporting its well-known association with GFR.

To summarize, CysC is a candidate biomarker of RRF in dialysis patients but potential confounders must be excluded in order to support its accuracy. Our study did not find a significant association of CysC levels with the metabolic profile and CVD events, but did suggest a tight correlation with RRF.
